# A novel translational model of atherosclerosis, the ex vivo pump-perfused amputated human limb model

**DOI:** 10.1038/s41598-024-67635-0

**Published:** 2024-07-27

**Authors:** Adam Hartley, Jonathan Afoke, Guiqing Liu, Samuel Owen, Reza Hajhosseiny, Kimberly Hassen, Prakash Punjabi, Dorian Haskard, Joseph Shalhoub, Ramzi Khamis

**Affiliations:** 1 Vascular Sciences Section, National Heart and Lung Institute, Imperial College, Hammersmith Hospital, London, UK; 2 Hammersmith Hospital, Imperial College Healthcare NHS Trust, London, UK; 3 Imperial College London and Imperial Vascular Unit, Imperial College Healthcare NHS Trust, Waller Unit, Mary Stanford Wing, St Mary’s Hospital, Praed Street, London, W2 1NY UK

**Keywords:** Atherosclerosis, Molecular targeting, Translational research, Atherosclerosis, Interventional cardiology, Cardiovascular models

## Abstract

The preclinical study of atherosclerosis has traditionally centred around the use of small animal models, translating to large animal models, prior to first-in-man studies. We propose to disrupt this paradigm by designing an ex vivo pump perfused human limb model. The novel model consists of taking a freshly amputated limb and incorporating it into an ex situ pump-perfused bypass system (akin to extracorporeal membrane oxygenation), circulating warmed, oxygenated blood. The circuit incorporates an introducer sheath and guiding catheter for intravascular imaging and X-ray angiography. Regular monitoring is performed using blood gas analysis, aiming for physiological parameters. The model maintains oxygen saturations > 99% for the length of perfusion (up to 6-h). Clinical grade X-ray angiography, intravascular ultrasound and optical coherence tomography have been successfully performed. Indocyanine green, a near-infrared fluorescent dye that localises to atherosclerotic plaque, has been injected into the system and left to circulate for 90-min. Fluorescence reflectance imaging of the dissected arterial bed confirmed uptake in areas of calcific atherosclerotic plaque on intravascular imaging. This is the first demonstration of an ex vivo pump-perfused “living” limb experimental model of atherosclerosis, which shows promise for future studies in translational interventional imaging and molecular targeting.

## Introduction

The traditional developmental pipeline of a novel molecular targeting agent, using atherosclerosis as an example, consists of the identification of an unmet clinical need, target discovery through interrogation and modulation of molecular cellular pathways in in vitro models, to in vivo translation with small animal models (e.g., mice and rats), prior to larger animal models, which are closer to human size and biology (e.g., rabbits and pigs), to demonstrate continued efficacy and absence of toxicity. Finally, following this, clinical studies must be conducted, ranging from small-scale first-in-man to large phase III clinical trials, testing against the current clinical standard. This translational pipeline is fraught with difficulties, with significant dropout of potential agents even after extensive and very promising in vivo testing, when a large amount of time, money and research effort will already have been invested.

The validity of animal models of atherosclerosis to patients with atherosclerotic cardiovascular disease in clinical practice has been questioned in this regard, with all models suffering from human translation applicability, cost, and ethical considerations. For example, transgenic mouse models, which are the most commonly utilised animal model, lack atherosclerotic lesion complexity, spontaneous plaque rupture or other atherothrombotic complications, rendering them models of atherogenesis rather than true cardiovascular disease^1^. Also, their small size means that intravascular experimental techniques cannot be performed. Larger animals with closer physiology to humans, where more clinically-orientated intravascular techniques can be employed, are more expensive to maintain, often more difficult to handle and have greater ethical considerations^2^.

As such, in this study we have designed, developed, and tested a novel translational model of atherosclerosis, centred around perfusion of an amputated human limb that would otherwise be clinical waste. This model will aim to provide a novel means of evaluating molecular targeting agents utilising human tissue, that should serve to both streamline translational pipelines and reduce our reliance on animals in preclinical atherosclerotic research.

Lower limbs amputated on clinical grounds will be used for the study, which de facto will have a significant burden of atherosclerosis, with chronic limb ischaemia representing the most common indication for amputation^3^. The limb will be incorporated into a bespoke extra-corporeal perfusion circuit that aims to maintain physiological parameters and facilitate sophisticated clinical and preclinical intravascular imaging techniques coupled with fluoroscopy. The limb will be perfused with warmed and oxygenated packed red cells mixed with balanced crystalloid via a modified oxygenator and a pulsatile pump. The pump will be set to mimic the cardiac output the limb would have received in situ. Measurement of pH, electrolytes, haemoglobin, lactate and oxygenation will be performed, with appropriate optimisation to ensure physiological parameters are maintained, as feasible.

## Results

**Figure 1:**
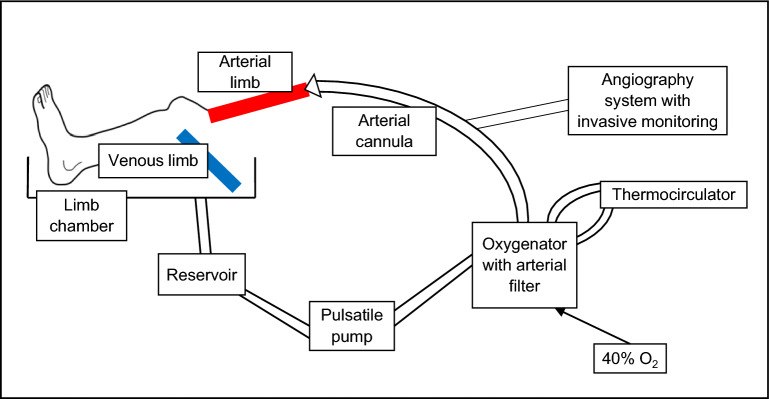
Schematic representation of the ex vivo pump-perfused amputated human limb model.

**Figure 2:**
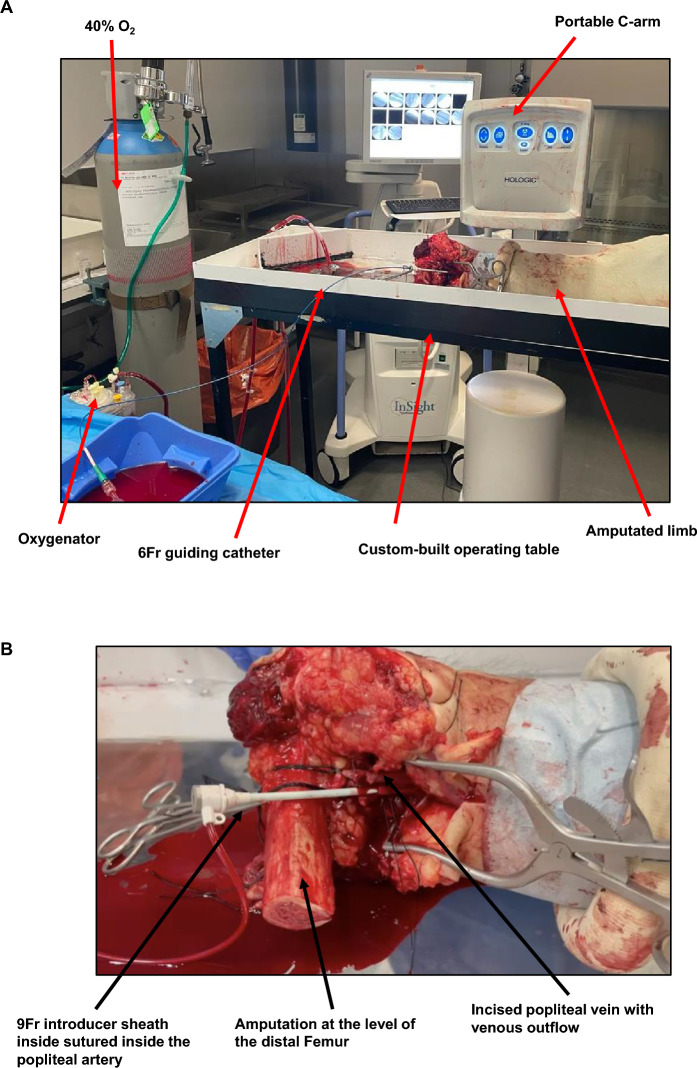
The amputated human limb model. (**A**) an overview of the model set-up, with fluoroscopic C-arm, oxygen supply and limb chamber demonstrated. (**B**) a close-up image of the perfusing sheath inside the popliteal artery, demonstrating passive outflow from the incised popliteal vein directly above the inflow sheath (black arrow).

The limb model has been successfully developed, antegradely perfusing amputated human lower limbs with warmed and oxygenated blood. Figure 1 provides a schematic representation of the limb circuit. After limb perfusion via cannulation of the femoral or popliteal artery (depending on length of resection), blood returns via incision of proximal veins and is collected passively into a reservoir. Blood is then pumped in a pulsatile fashion through an oxygenator and arterial filter prior to returning the limb via the arterial cannula. In parallel, a thermocirculator provides a warming circuit to heat the perfusate to 37 °C prior to re-circulation. This model allows the passage of an introducer sheath and guiding catheter for intravascular imaging and X-ray angiography, and has been incorporated into an experimental catheter laboratory. Figure 2 provides images of the limb model during use.

### Limb perfusion

The perfusate comprised either expired packed red cells and balanced crystalloid in a 1:1 ratio. Sodium heparin was added to the perfusate to limit haemostasis. The pump was set at 60 systole %/40 diastole % (to equate to an approximated clinical blood pressure) and at a pump output of 15 mL per stroke (to represent approximately 10% of clinical stroke volume), with an average pump rate of 70 per minute (equating to a heart rate of 70 beats per minute). Thus, total pump output, or ‘cardiac output’ to the limb was estimated at 1050 mL/min. Circuit flow (mL/min) was approximated to 6–10% of donor estimated cardiac output (pulse pressure/[(systolic blood pressure + diastolic blood pressure) × heart rate]), which is an approximation of the blood flow a leg would receive in vivo.

Monitoring for satisfactory limb perfusion was performed using continuous pulse oximetry, hourly peripheral temperature and post-limb perfusate testing every hour for pH, oxygen saturation, lactate, glucose, bicarbonate, sodium, potassium, and haemoglobin concentrations. The perfusate was adjusted according to these parameters with sodium chloride containing 40 mmol potassium, 1.26% sodium bicarbonate and 10% dextrose in solution as required, aiming for physiological electrolyte normal ranges. Haemoglobin concentration was targeted at 40–60 g/L, with further packed red cells/crystalloid added as required. This haemoglobin target range was based on prior studies with upper limb perfusion, where this lower than physiologic level resulted in less vascular congestion^4^. Oxygen saturations were targeted at > 99%. In addition, continuous haemodynamic monitoring was performed using beat-to-beat invasive blood pressure, heart rate and oxygen saturations.

**Figure 3:**
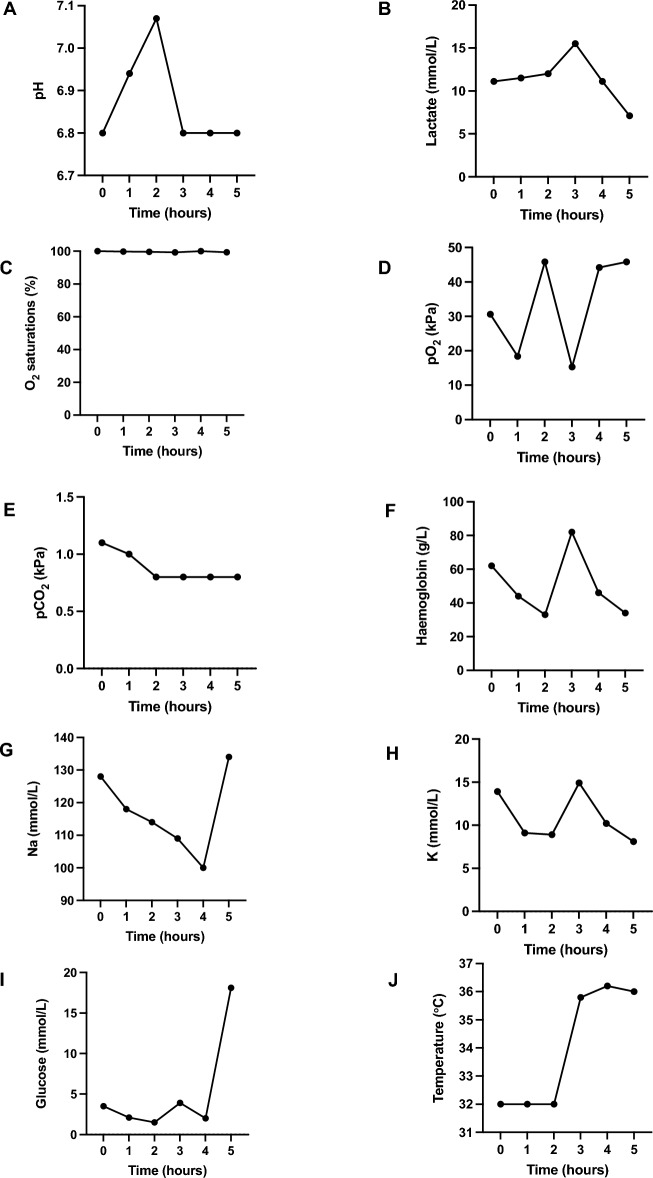
Physiological parameters measured during an example limb perfusion set-up. (**A**) pH. (**B**) lactate (mmol/L). (**C**) oxygen saturations (%). (**D**) partial pressure of oxygen (kPa). (**E**) partial pressure of carbon dioxide (kPa). (**F**) haemoglobin (g/L). (**G**) sodium (mmol/L). (**H**) potassium (mmol/L). (**I**) glucose (mmol/L). (**J**) temperature (°C).

Figure 3 provides an overview of the measured parameters during example limb perfusion. Gas exchange is excellent, with the partial pressure of oxygen maintaining > 15 kPa and oxygen saturations > 99% throughout. In addition, carbon dioxide levels were very low at < 1.5 kPa. Haemoglobin stayed around the target range of 40–60 g/L throughout. The skin temperature of the limb did take a significant length of time to rise towards normothermia, likely due to the cold ischaemic time on ice packs during transit prior to perfusion. Unfortunately, despite exchanging blood volume with fresh crystalloid and packed red cells, it was not possible during this experiment to generate a non-acidotic pH or suppress the lactataemia.

### Angiography and intravascular imaging

**Figure 4:**
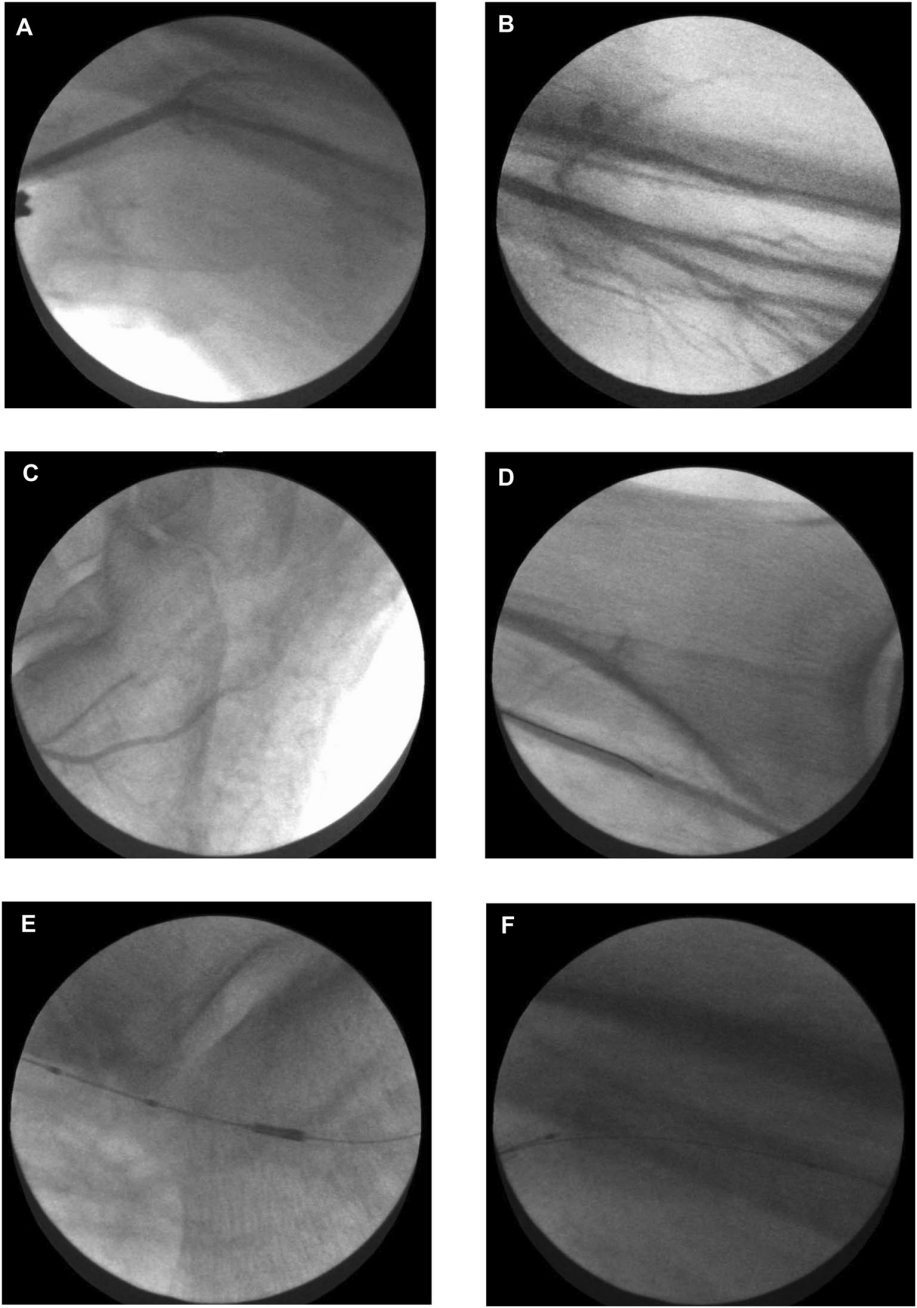
Angiography of the amputated human limb model. (**A**) Perfusion of an above knee amputation via the popliteal artery, with an angiogram taken in posterior-anterior projection with the injection of iodinated contrast (Omnipaque 300, GE Healthcare, Illinois, USA) through a 6French multipurpose guiding catheter (MB1 Launcher, Medtronic, Ireland) at knee level. (**B**) Angiogram at the below knee level, demonstrating the anterior tibial artery (top), tibioperoneal trunk (bottom left) bifurcating into the posterior tibial and peroneal arteries. (**C**) Angiogram at the level of the foot, demonstrating the medial (top) and lateral (bottom) plantar arteries, arising from the posterior tibial artery. (**D**) A coronary 0.014″ guidewire (ChoICE PT Extra Support, Boston Scientific, Massachusetts, USA) was advanced within posterior tibial artery to level of ankle. (**E**) An intravascular ultrasound catheter (Volcano Eagle Eye Platinum—Philips, Amsterdam, Netherlands) was advanced to level of ankle within the posterior tibial artery. (**F**) An optical coherence tomography catheter (Dragonfly Optis—Abbott Cardiovascular, Illinois, USA) was advanced to the proximal posterior tibial artery.

Following the establishment of limb perfusion, angiography is performed in each limb, confirming arterial perfusion, and identifying areas of atherosclerosis. Figure 4 and corresponding Movies 1–5 provide example fluoroscopic acquisitions using iodinated contrast obtained in one limb model obtained from an individual who was a prior smoker with diabetes mellitus, hypertension and hypercholesterolaemia; demonstrating successful intubation of the popliteal artery at knee-level (A); just distal to the popliteal trifurcation below the knee, with anterior tibial, posterior tibial and peroneal branches shown (B); at foot-level with plantar arterial branches of the posterior tibial artery demonstrated (C). Following successful wiring of the vessel of interest (posterior tibial artery) (D) using a 0.014″ coronary guidewire, intravascular imaging runs were performed, with intravascular ultrasound (IVUS) (E) and optical coherence tomography (OCT) (F) catheters passed over-the-wire.

**Figure 5:**
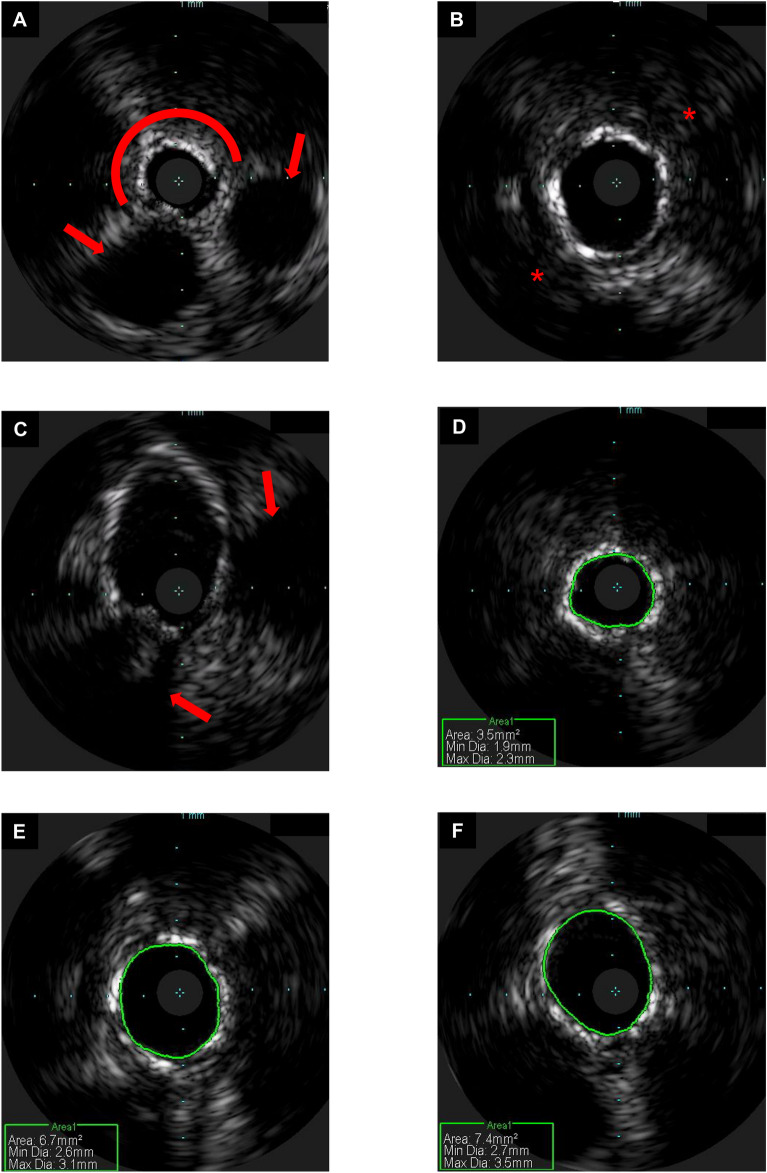
Intravascular imaging of the amputated human limb model using intravascular ultrasound. Intravascular ultrasound study of the amputated human limb model performed using the Eagle Eye Platinum catheter (Volcano, Philips, Amsterdam, Netherlands). (**A**) Still frame of the distal posterior tibial artery, demonstrating superficial calcification (7 o’clock to 3 o’clock, highlighted by red arc) without significant luminal obstructive disease. There are surrounding extraluminal vessels (highlighted by red arrows), which likely represent posterior tibial venous branches veins running alongside the posterior tibial artery. (**B**) Still frame of the mid posterior tibial artery, demonstrating concentric calcification, with loss of signal penetration beyond the calcium deposits (highlighted by red *). (**C**) Still frame demonstrating the tibioperoneal bifurcation, as the posterior tibial artery joins the peroneal artery (highlighted by red arrows). (**D**–**F**) Minimal lumen area measurements in the distal (**D**), mid (**E**) and proximal (**F**) posterior tibial artery. The interval between crosshair indicators represents 1 mm.

**Figure 6:**
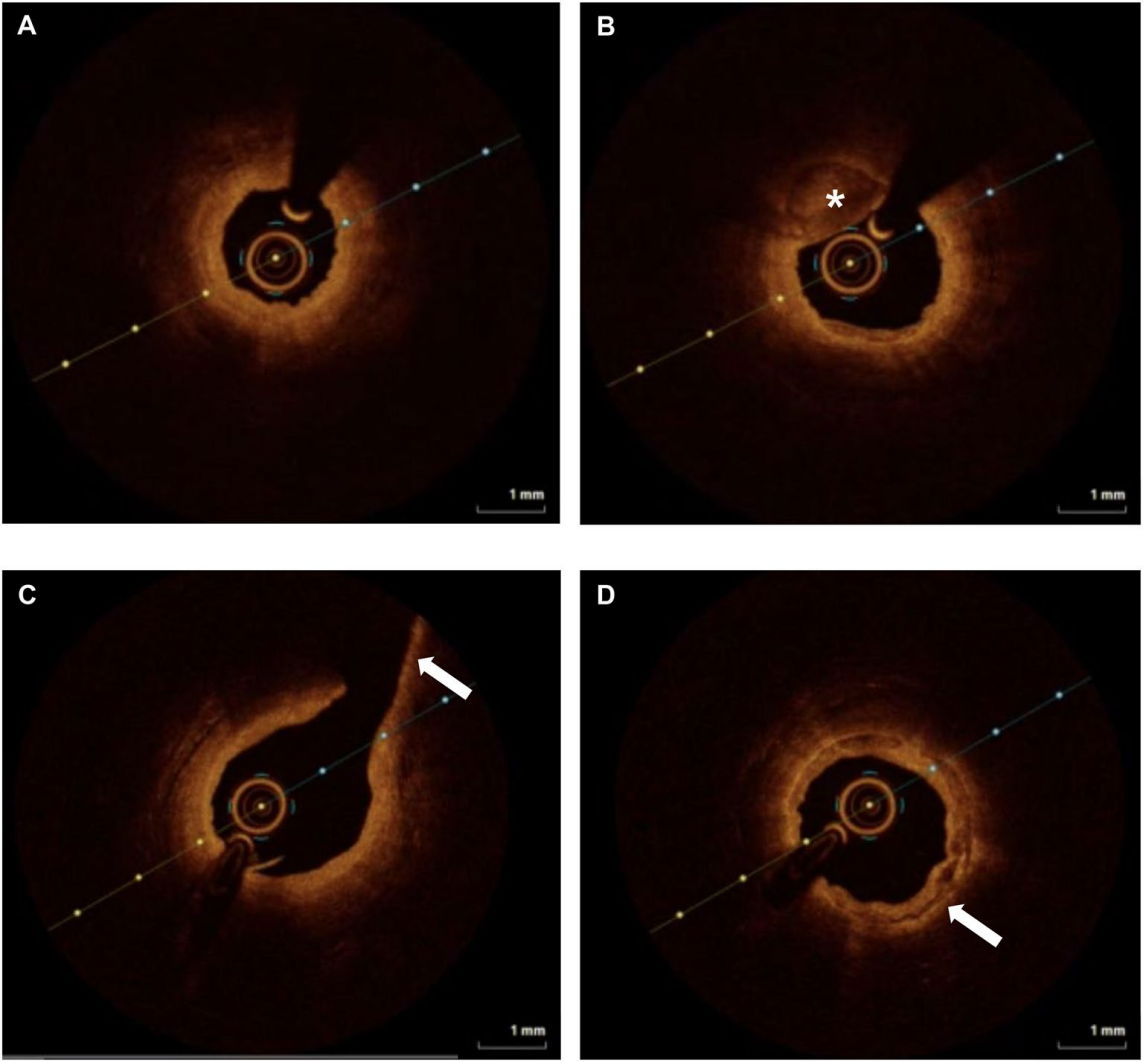
Intravascular imaging of the amputated human limb model using optical coherence tomography. Optical coherence tomography study of the amputated human limb model performed using the Dragonfly Optis catheter (Abbott Cardiovascular, Illinois, USA). Injection of iodinated contrast was used to generate a blood-free field. (**A**) still frame of the distal posterior tibial artery, demonstrating concentric fibrous plaque with a high backscatter and relatively homogenous signal. (**B**) Still frame of the mid posterior tibial artery, demonstrating a fibrous calcific plaque with a large area of calcification (indicated by *), appearing as a signal-poor well demarcated region with clear borders and no dramatic signal drop-out. (**C**) Still frame demonstrating the tibioperoneal bifurcation, as the posterior tibial artery joins the peroneal artery (highlighted by white arrow). (**D**) Still frame of the popliteal artery showing concentric fibrous calcific plaque (highlighted by white arrow). The interval between crosshair indicators represents 1 mm.

Intravascular imaging plays an important role in clinical interventional cardiology, to both assess atherosclerotic lesion morphology prior to possible percutaneous coronary intervention (PCI) and evaluate the post-PCI result. Accordingly, intravascular imaging has an important role in translational research, permitting the detailed analysis of effective molecular targeting to varying atherosclerotic plaque characteristics. IVUS is a commonplace tool in modern clinical cardiac catheter laboratories, that utilises an ultrasonic transducer at the distal catheter tip to visualise the arterial wall with a good resolution of 100 µm^5^. OCT is a near-infrared light-based imaging modality that generates high-resolution cross-sectional images of the vessel wall, with a resolution of 10–15 µm^6^. In the experimental limb model, IVUS and OCT imaging was performed of the vascular bed, from popliteal artery to distal posterior tibial artery of the same limb shown in Fig. 4. Example images and movies acquired using these tools are displayed in Figs. 5 and 6, as well as corresponding Movies 6 and 7. Figs. 5 and 6 and Movies 6/7 demonstrate evidence of atherosclerotic plaques that could not be readily appreciated angiographically. The obtained intravascular imaging is of clinical calibre and is easily interpretable by a trained operator.

### Evaluation of a near-infrared atherosclerotic molecular targeting agent

As a proof-of-principle of utilising the limb model for the assessment of a molecular targeting atherosclerosis agent, indocyanine green was tested. Indocyanine green is a near-infrared agent (excitation 780/emission 830 nm), which targets lipid and macrophages in areas of increased endothelial permeability (atherosclerotic plaques). Moreover, indocyanine green is readily available as it is used clinically with laser ophthalmoscopy for the evaluation of the retinochoroidal circulation.

**Figure 7:**
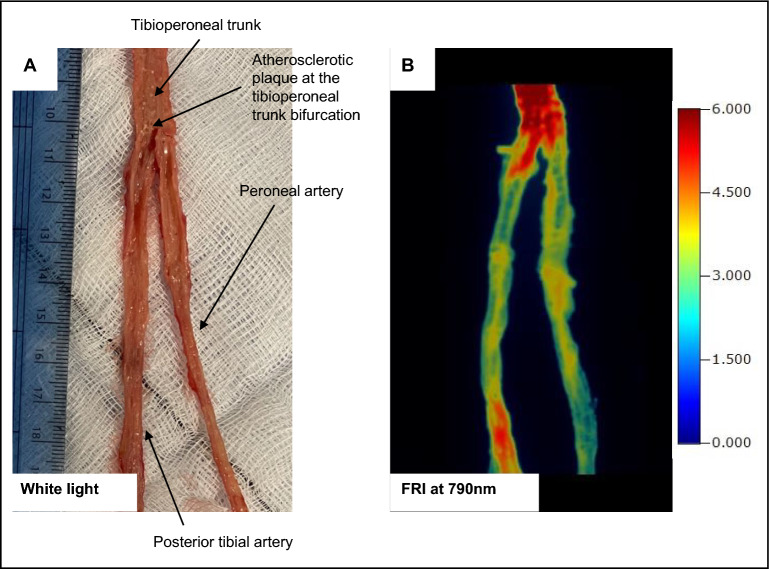
Targeting atherosclerosis near-infrared fluorescence imaging of atherosclerosis using the ex vivo pump-perfused amputated human limb model. Indocyanine green (2 mg/kg) was injected post-limb and left to circulate in the perfusion system for 90-min. Following this, the limb was flushed with fresh crystalloid and the perfused arterial tree dissected out, opened en face (lumen facing upwards) and imaged under white light. The tibioperoneal trunk bifurcation into posterior tibial artery on the left and peroneal artery on the right is shown (**A**). Fluorescence reflectance imaging was then performed using the FMT 4000 at 790 nm (**B**). Atherosclerotic plaque at the tibioperoneal trunk bifurcation is highlighted in (**A**) that also demonstrates increased indocyanine green uptake in (**B**). Scale bar in (**A**) is in cm. Scale bar in (**B**) is in counts/ energy, ranging from blue (low, 0) to red (high, 6.0).

Indocyanine green at a dose of 2 mg/kg (limb weight) was injected post-limb (akin to intravenous injection) and left to circulate within the perfusion system. Indocyanine green usually has a human plasma half-life of 3–5 min, with hepatic excretion. Of course, in the limb model there is no liver metabolism; thus, the agent will remain in circulation until the perfusate is changed. In an experimental in vivo human carotid study utilising indocyanine green to visualise carotid plaques, the average time from intravenous injection to ex vivo imaging was 99 min^7^. Accordingly, 90-min after injection, limb perfusion was ceased, and the system drained of all perfusate. The limb was then perfused with fresh crystalloid to flush any residue or unbound indocyanine green and therefore reduce background fluorescent signal. Next, the perfused arterial tissue was dissected and opened en face, prior to performing fluorescence reflectance imaging (FRI), shown in Fig. 7. This displays the dissected tibioperoneal trunk bifurcating into posterior tibial and peroneal arteries, imaged under white light in (A) and using FRI in (B). There is increased fluorescence signal in the tibioperoneal artery proximal to the bifurcation, which is a predilection site for atherosclerosis, as well as in the posterior tibial artery. The area of high intensity fluorescence towards the distal end of the posterior tibial artery section corresponds to regions of circumferential calcification seen on both IVUS and OCT in Figs. 5 and 6.

## Discussion

This study details the design, development and validation of a novel experimental model of atherosclerosis.

The benefits of this novel amputated human limb model of atherosclerosis lie in that it could reduce our dependency on animal models of atherosclerosis in preclinical research, whilst at the same time also improving applicability of the preclinical models to humans, thus optimising clinical relevance, and hopefully increasing the efficiency of clinical translation. Although establishing the limb perfusion circuit was initially expensive, once set up, there is a possibility that this model may be cost saving, versus breeding/purchasing, feeding and housing animals (especially large species), as well as any necessary procedures, such as balloon iliac injury in the case of the rabbit model of atherosclerosis.

Rabbit models of experimental atherosclerosis are common, owing to their relatively inexpensive maintenance and easy handling. Experimental use is declining however, likely related to the ready commercial availability of cheaper and more acceptable transgenic mouse models. Rabbit models include Watanabe heritable hyperlipidaemic rabbits, which develop significant hypercholesterolaemia and hypertriglyceridaemia accompanied by widespread atherosclerotic lesions with a normal diet^8^. Other approaches include New Zealand White rabbits fed a high-fat diet and more recently, Apolipoprotein E-deficient rabbits have been generated using genome editing^9^. Rabbit models develop largely foam cell atherosclerotic lesions, rather than the fibrous lesions in mice and more complex mixed lesions in humans. Rabbits are obviously larger in size than mice, and therefore iliac vessel cannulation is possible, permitting catheter-based intravascular imaging of the aorta, but not the coronary arteries. This has been harnessed to generate a novel model of atherosclerosis, whereby catheter-directed balloon injury is performed to disrupt the endothelium of the iliac vessels, resulting in rapid neointimal thickening, lipid accumulation, smooth muscle cell infiltration and foam cell generation. This creates atherosclerotic lesions in an accelerated fashion (within 2-weeks), much faster than the four to eight months that would be required otherwise^10^. Rabbit lipoprotein metabolism is closer to human than mouse lipoprotein metabolism, however there are important differences. For example, rabbits lack Apolipoprotein A-II (an important component of High-Density Lipoprotein in humans) and have low levels of hepatic lipase^11^. Again, different to humans, rabbits develop significant atherosclerotic lesions in the aortic arch and descending thoracic aorta^2^.

Larger animal models of experimental atherosclerosis are principally pigs and non-human primates. The advantages of using these larger species over smaller species, are that more complex intravascular catheter-based techniques can be employed, owing to their increased size and more human-like cardiovascular anatomy.

Pigs are the most used larger species for experimental atherosclerosis modelling, owing to their human-like anatomy, closer genetic resemblance to humans and similar atherosclerosis predilection sites, in the coronary, abdominal aorta and iliofemoral arteries. However, atherosclerotic plaques in porcine models often do not advance to become more complex lesions, at least in a reasonable experimental timescale. Furthermore, spontaneous plaque rupture and thrombosis is rare. Pig models consist of lines with natural mutations in the Apolipoprotein B and Low-Density Lipoprotein-Receptor genes, diabetic hypercholesterolaemic pigs, as well as genetically engineered strains, for example, Low-Density Lipoprotein-Receptor knock-out. In contrast to mice and rabbits, pigs are more expensive to maintain and more difficult to handle^2^. Porcine models however have been successfully utilised to study atherosclerotic plaque development in response to disturbed coronary flow using clinical calibre in vivo intravascular imaging^12^, model ischaemia–reperfusion injury and restenosis following angioplasty, as well as being used in stent development^13^.

Non-human primates, for example, cynomolgous and rhesus monkeys, develop atherosclerosis on high-fat diets, and can be useful experimental models for human atherosclerosis. However, they are used very infrequently, owing to the necessary level of regulation, expense, ethical considerations, as well as high level of specialised training required. Nonetheless, non-human primate studies have been utilised to study psychosocial and behavioural aspects of atherosclerosis^13^.

Maintenance of a physiological environment is desirable to allow the experimental set-up to continue running for a prolonged period without limb decomposition, to assess probe binding and localisation at a timepoint after initial administration, simulating intravenous construct injection. As an example, ex situ perfusion of an amputated human upper limb from brain-dead organ donors has been successfully performed for 24-h (n = 5), in an attempt to prolong allograft storage times for transportation and potential transplantation. Using a similar experimental circuit design, neuromuscular electrical stimulation resulted in appropriate contractile responses throughout perfusion and histological examination post-perfusion demonstrated no evidence of myocyte injury^4^. Unfortunately, despite exchanging blood volume with fresh crystalloid and packed red cells, it has not been possible to generate a non-acidotic pH or suppress the lactataemia in this model. This is likely a result of a degree of muscle breakdown and anaerobic respiration of the heavily diseased amputated limb tissue, as well as the lower pH and raised lactate concentration of stored packed red cells^14^. However, this raises the unique possibility of utilising the limb model to study the effects of various physiological stressors that may promote plaque rupture within human vessels.

Achieving physiological levels of sodium, potassium and glucose was problematic, possibly due to the large swings in electrolyte/ glucose levels induced by the addition of large volumes of crystalloids within a smaller circulating volume (the circuit volume is approximately 2 L, compared to 5 L in an adult male). Using glucose as an example, a very large jump in glucose concentration is seen at 5-h in Fig. 3, due to blood sampling occurring soon after the administration of 10% dextrose solution. Transfusion-related hyperkalaemia is a well identified phenomenon, especially with blood that has been stored for longer periods, due to reduced cellular function and potassium leakage into the extracellular space^15^. The red cells used in this experiment will have been stored for a prolonged period prior to use, and thus will carry a significant potassium load. Moreover, as above, given the smaller circulating volume of the limb circuit and packed red cells forming half the circulating volume, any changes in electrolyte levels are magnified. Indeed, transfusion-related hyperkalaemia has been noted to be more problematic in neonatal and paediatric populations^16^.

Important limitations include the heavily atherosclerotic nature of the amputated limbs, which will restrict the ability to achieve optimal physiological conditions for limb perfusion. Despite the significant disease burden, successful perfusion has always been achieved, likely due to the preservation of viable tissue within resection margins during the amputation. In addition, although animal models are often used to demonstrate targeting probe function, data on harm or toxicity is also generated. This will not be possible with the limb model. Likewise, evaluating pharmacokinetics will not be possible with the limb model, nor the assessment of activatable or pro-drug compounds. Moreover, animal models are routinely used to assess therapeutic efficacy of an investigational agent: this will be difficult to achieve with the limb model, without the ability to run the model for sufficient time to allow a therapeutic agent time to demonstrate efficacy. Additionally, although this is human-based and great efforts have gone to optimise the model within physiological parameters, it remains an artificial system, where clinical applicability and relevance will still be questioned. A further consideration is the relevance of peripheral atherosclerotic plaques to coronary or cerebrovascular atherosclerotic disease. Peripheral atherosclerosis tends to have greater burden of calcification, including vascular medial calcification. As a result, plaque rupture of peripheral plaques is less common, and occlusive clinical disease is more often the result of embolic phenomena or in situ thrombosis^17^.

There are further considerations regarding tissue stability for the use of this model in other research avenues. For example, we have not utilised prolonged perfusion times and demonstrated continuing limb viability. However, as discussed above, high levels of oxygenation in the venous perfusate were achieved across all experimental time points, as well as successful tissue warming following perfusion and a declining lactate concentration, all suggesting potential model stability. On a practical level, once perfusion has been established, the model requires little input, aside from perfusate sampling and adjusting conditions accordingly. One option to experimentally evaluate model viability would be to obtain both proximal and distal limb skin tissue biopsies and carry out a cell viability or metabolic activity assay at varying timepoints, with the aim of demonstrating continuing model stability of both tissue samples. Another potential utility of this model would be in evaluating vascular physiological functions in response to various stimuli, potentially with invasive physiological assessments, as performed routinely in clinical interventional cardiology practice. However, this model may not be ideal to evaluate vascular physiology, given the artificial nature of the perfusion circuit, with potential confounding cellular effects of altered flow dynamics as well as the acidotic cellular environment.

Moving forwards, challenges with this novel experimental model will include further optimisation of perfusion conditions, aiming to normalise the cellular environment for both pH and electrolyte balance. Performing prolonged perfusion experiments (> 24 h) with demonstration of continued tissue viability will also be an important next step. Once these have been established, testing novel molecular targeting agents on human tissue, with detection via catheter based near-infrared fluoroscopic techniques as well as other non-invasive imaging modalities, will be an exciting prospect.

Successful ex situ molecular targeting of human atherosclerotic plaque has been demonstrated for the first time, through this novel translational model of atherosclerosis, using clinical calibre intravascular imaging with IVUS and OCT to permit plaque signal colocalisation. Potential future uses include the preclinical testing of developmental molecular probes, as well as trialling new intravascular imaging catheters, without the requirement for animal models and with the hope of more streamlined bench to bedside translation.

## Methods

### Ethical permissions

Ethical permission for the collection of, and research on, human clinical tissue for this study was granted by the Imperial College Healthcare Tissue Bank (REC Wales 17/WA/0161), as a formal subcollection (CAR_RK_17_070). No additional clinical samples were required or obtained for this study. All methods were performed in accordance with necessary institutional guidelines and regulations.

Patients were approached to donate tissue to the study following screening by the referring vascular surgical team and routine clinical pre-amputation counselling, arranged from pre-operative clinic. All participants received the appropriate patient information leaflet for use of leftover tissue for research purposes, as per the Imperial College Healthcare Tissue Bank, prior to the consenting process. All patients provided written informed consent for tissue collection at the time of consenting for the operation itself.

### Ex situ perfusion system

A custom-made radiolucent operating table was designed that incorporated a decline and funnel to facilitate venous blood return back into the bypass circuit. The funnel was connected via ¼’’ tubing (Tygon Tubing, Harvard Apparatus, Cambridge, UK) to the venous inlet of a modified oxygenator with integrated arterial filter (CAPIOX Baby FX05RW, Terumo, Surrey, UK), allowing the venous reservoir to fill passively under gravity. The arterial filter served to remove any clots or debris generated within the perfusion system. The reservoir outlet was connected to the inlet of a pulsatile blood pump, with ball valves and smooth flow to minimise haemolysis (55-3321 pulsatile blood pump for dogs/ monkeys, Harvard Apparatus, Cambridge, UK), through a ¼″–½″ adapter (Harvard Apparatus, Cambridge, UK) and ½″ tubing. The pump outlet was connected through a ½″–¼″ adapter to ¼″ tubing back to the arterial inlet of the oxygenator. The oxygenator was supplied with a sweep gas of 40% oxygen: air mixture via ¼″ tubing. A parallel circuit supplying warmed water to the oxygenator at 37 °C was set up using a thermocirculator (TC120, Grant Instruments, Cambridge, UK), connected via ½″ tubing. The arterial outlet port of the oxygenator was connected via ¼″ tubing to one arm of a Y-connector (Harvard Apparatus, Cambridge, UK). Further ¼″ tubing incorporating a femoral sheath (9French Input Introducer Sheath, Medtronic, Dublin, Ireland) was connected to the other arm. The Y-connecter was then joined via further ¼″ tubing to a femoral arterial cannula (Medtronic EOPA Elongated One-Piece Arterial cannula, Dublin, Ireland), supplying the amputated limb with warmed and oxygenated perfusate.

### Limb procurement

All patients undergoing elective planned limb amputations were eligible to donate tissue to the study. Limbs were collected immediately from vascular operating theatre and flushed with heparinised saline to remove clots and prevent in situ thrombosis. Ligatures were tied around the main proximal artery that would be used for antegrade perfusion, as well as the large proximal veins for outflow, locking the heparinised saline within the vascular bed.

### Limb perfusion

Firstly, the arterial cannula was sutured into the femoral/popliteal artery (depending on length of resection), and corresponding veins incised to facilitate venous return. Following establishment of the perfusion circuit, the limb was thoroughly flushed via the pulsatile pump with heparinised saline to remove any in situ clot. The system was then drained to remove the saline wash and exchanged for perfusate. The perfusate comprised either expired clinical-grade or research-grade packed red cells (Non-Clinical Issue National Health Service Blood and Transplant Service, Colindale, UK) and balanced crystalloid (Plasma-Lyte 148 [pH 7.4], Baxter, Berkshire, UK) in a 1:1 ratio. Packed red cells were used for their oxygen carrying capacity, rather than an acellular perfusate. Sodium heparin was added to the perfusate, at a concentration of 3,000 units/kg in 0.9% sodium chloride, to limit haemostasis.

Monitoring for satisfactory limb perfusion was performed using continuous pulse oximetry (AVAX, Contec Medical Systems, China); hourly peripheral temperature of the skin on the sole (TempIR, USA); and post-limb perfusate testing every hour for pH, oxygen saturation, lactate, glucose, bicarbonate, sodium, potassium, and haemoglobin concentrations (GEM 4000, Werfen UK, Cheshire, UK). In addition, continuous haemodynamic monitoring was performed using a S/5 Compact Anaesthesia Monitor with beat-to-beat invasive blood pressure, heart rate and oxygen saturation modules (M-ESTR module) (Datex-Ohmeda, Wisconsin, USA).

### Imaging

Interventional cardiac guide catheters (for example, a 6French multipurpose MB1 Launcher guiding catheter [Medtronic, Dublin, Ireland]) were introduced over a J-tipped 0.035″ wire to the artery of study and connected to a 3-port standard angiography manifold, that permits injection of iodinated contrast, invasive monitoring and a port for administration of other agents. Omnipaque 300 (GE Healthcare, Illinois, USA) was used as the contrast agent for angiographic studies. X-ray fluoroscopy was performed using a portable C-arm (Fluoroscan Insite FD Mini C-arm Extremities Imaging System, Hologic, Manchester, UK). Following this, 0.014″ coronary guidewires, such as a Runthrough NS Extra Floppy (Terumo, Tokyo, Japan), could be manipulated to the distal arterial bed using conventional interventional techniques. Over this wire, intravascular imaging catheters could be passed distal to the area of interest and manual/automated pullback performed. Readily available imaging systems included IVUS (Volcano Eagle Eye Platinum, Philips, Amsterdam, Netherlands) and OCT (Dragonfly Optis, Abbott Cardiovascular, Illinois, USA). IVUS was performed with manual pullback at a rate of 1 mm/s, whilst OCT was performed with an automated pullback of 20 mm/s.

### Supplementary Information

Supplementary Video 1.

Supplementary Video 2.

Supplementary Video 3.

Supplementary Video 4.

Supplementary Video 5.

Supplementary Video 6.

Supplementary Video 7.

Supplementary Information.

## Data Availability

The data that support the findings of this study are available from the corresponding author, RK, upon reasonable request.
