# Supplementary material for: A novel translational model of atherosclerosis, the ex vivo pump-perfused amputated human limb model
Source: Sci Rep. 2024 Jul 27;14:17244. doi: 10.1038/s41598-024-67635-0 (PMC11282226; doi:10.1038/s41598-024-67635-0)
Supplement: Supplementary file 8 — Supplementary Information. [file 41598_2024_67635_MOESM8_ESM.docx]

**Supplementary Materials**

**Movie Legends**

**Movie 1** – Lower limb angiogram from popliteal artery to plantar branches.

**Movie 2** – Advancing the 0.014” intracoronary guidewire within the posterior tibial artery.

**Movie 3** – Lower limb angiogram with the 0.014” intracoronary guidewire in situ.

**Movie 4** – Intravascular ultrasound catheter manual pullback.

**Movie 5** – Optical coherence tomography catheter automated pullback.

**Movie 6** – Intravascular ultrasound run from distal to proximal vascular bed.

**Movie 7** – Optical coherence tomography run from distal to proximal vascular bed.
